# Synergistic Photomagnetic and Photomechanical Dynamics in a Dysprosium‐Based Smart Molecule

**DOI:** 10.1002/advs.202509088

**Published:** 2025-07-13

**Authors:** Yu‐Han Wang, Shi‐Kun Yan, Shuai Liang, Yan‐Rui Zhao, Jin Zhang, Guo‐Ming Wang, Ji‐Xiang Hu

**Affiliations:** ^1^ Department College of Chemistry and Chemical Engineering Qingdao University Qingdao Shandong 266071 China

**Keywords:** cycloaddition, photo/thermoswitching, photomechanical motion, single‐molecule magnets

## Abstract

Converging photomagnetic switching and photomechanical functionalities in molecular materials is critical for next‐generation adaptive technologies, yet challenged by competing demands for rigid coordination environments to suppress quantum tunneling in single‐molecule magnets (SMMs) and flexible frameworks to enable macroscopic motion. Herein, photoactive 9‐anthracenecarboxylic acid (HAC) is integrated into a binuclear dysprosium(III) complex, (**1**), which simultaneously exhibits dual light‐responsive behaviors of both SMMs and macroscopic mechanical motion. The 365 nm light irradiation induces [4+4] photocycloaddition of anthracene units in **1**, driving a single‐crystal‐to‐single‐crystal transformation to a 1D coordination polymer [Dy₂(HAC)₂(DMF)₂(AC)₆]n (**1A**). This structural reorganization boosts axial magnetic anisotropy, unlocking SMM behavior with a butterfly‐shaped hysteresis loop, whereas thermal annealing regenerates the pristine structure and quenches magnetic bistability. Notably, anisotropic lattice strain from cycloaddition induces macroscopic photomechanical motions, triggering macroscopic torsional dynamics in bulk crystals and pronounced flexural deformations in thin film architectures. This work pioneers the synergistic modulation of magnetic and mechanical properties through light‐driven structural dynamics in a single‐component system, establishing a fundamental platform for developing intelligent materials with integrated opto‐magneto‐mechanical functionalities for adaptive sensing and actuation technologies.

## Graphical abstract

Photoactive 9‐anthracenecarboxylic acid integrates into a Dy(III) complex enables dual light‐response. UV irradiation triggers anthracene [4+4] cycloaddition, driving a single‐crystal‐to‐single‐crystal transformation to coordination polymer **1A**. This structural change enhances axial anisotropy, activating SMM behavior with butterfly hysteresis. Concurrent anisotropic strain induces macroscopic photomechanical motions: torsional dynamics in bulk crystals and flexural bending in thin films.

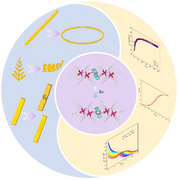

## 1 Introduction

The pursuit of next‐generation adaptive technologies has stimulated growing interest in molecular materials that combine photomagnetic switching with photomechanical actuation.^[^ ^1^, ^2^, ^3^, ^4^, ^5^ ^]^ These dual‐responsive systems, capable of simultaneous spin state modulation and mechanical deformation under photonic excitation, present unique opportunities for developing multifunctional platforms that bridge quantum spin phenomena with macroscopic actuation. This emerging field builds upon two well‐established research trajectories: photomagnets exhibiting the variations of magnitude and direction in magnetization under specific optical conditions^[^ ^6^, ^7^, ^8^, ^9^, ^10^, ^11^, ^12^, ^13^ ^]^ and photomechanical crystals capable of transducing photon energy into macroscopic motion via tailored photochemical pathways.^[^ ^14^, ^15^, ^16^, ^17^, ^18^, ^19^ ^]^ Recent advances in photoinduced molecular magnets, especially single‐molecule magnets (SMMs), have elucidated design principles for achieving magnetic bistability through ligand‐field engineering and electron transfer/spin transitions, positioning these materials as promising candidates for high‐density memory devices and quantum logic elements.^[^ ^20^, ^21^, ^22^, ^23^, ^24^, ^25^, ^26^ ^]^ In parallel, concurrent progress in photomechanical systems has demonstrated precise control over crystalline actuation through strategically engineered [4+4]/[2+2] cycloadditions and trans‐cis isomerizations, enabling applications ranging from soft robotics to energy‐harvesting devices.^[^ ^27^, ^28^, ^29^, ^30^, ^31^, ^32^ ^]^ The structural adaptability required for these photoinduced phase transitions has enabled remarkable demonstrations of crystalline actuators and energy transduction systems. Integrating these two distinct functionalities, photomagnetic bistability and photoinduced mechanical deformation, into a unified platform could enable innovations in magneto‐optical actuators, phototunable spintronic devices, or self‐adaptive systems responsive to multiple stimuli. Recent experimental advances showcase this potential through: Poshakinskiy's demonstration of optically detected spin‐mechanical resonances in SiC membranes;^[^ ^33^ ^]^ Yang et al.’s light‐actuated magnetic hydrogel actuator via photothermal‐induced hydration modulation;^[^ ^34^ ^]^ and Li group's systematic analysis highlighting advances in flexible magnetosensitive families.^[^ ^35^ ^]^ However, achieving such synergy remains a formidable challenge, due to the conflicting design principles: SMMs require rigid, symmetric coordination environments to suppress QTM, whereas photomechanical activity demands structural flexibility and dynamic bond reorganization to accommodate photoinduced strain,^[^ ^36^, ^37^, ^38^, ^39^ ^]^ the combination of light‐induced SMM behavior and photomechanical motion in a single molecule has not been achieved for now.

Given the structural designability and performance tunability, coordination chemistry in recent advances has shown that sensible molecular assembly engineering can reconcile these opposing requirements to achieve the assembly of bifunctional molecules. Anthracene‐based ligands are particularly attractive due to their ability to undergo reversible [4+4] cycloaddition under light irradiation, which induces significant structural reorganization and molecular strain, providing an ideal scaffold for introducing photoresponsivity.^[^ ^40^, ^41^, ^42^, ^43^, ^44^ ^]^ When combined with lanthanide ions like dysprosium (Dy^III^), these systems offer a unique platform to explore the light‐triggered SMM behavior and photomechanical motion, which hold potential for applications in high‐density data storage, molecular switches, and soft robotics. In this regard, the light‐responsive single‐molecule magnets and photomechanical responsive molecules constructed by anthracenecarboxylic acid have been respectively studied.^[^ ^45^, ^46^, ^47^ ^]^ However, such dual‐functional materials are still not achieved despite the broad prospects, primarily due to difficulties in synchronously actuating SMM behavior and preserving mechanical properties during photochemical reactions.

Motivated by this background, we constructed a new binuclear compound, [Dy_2_ (HAC)_2_(DMF)_2_(AC)_6_] (**1**, HAC = 9‐anthracene carboxylic acid, DMF = N, N‐dimethylformamide), that simultaneously exhibits light‐induced SMM behavior and photomechanical motion. Upon light irradiation with a 365 nm fiber, **1** underwent a remarkable single‐crystal‐to‐single‐crystal (SC‐SC) transformation driven by the photocycloaddition of adjacent AC ligands, leading to single‐chain architectures [Dy_2_(DMF)_2_(HAC)_2_(AC)_6_]n (**1A**). The photocycloaddition product profoundly modified the ligand field and magnetic anisotropy, thereby eliciting the SMM phenomenon with a butterfly‐shaped hysteresis loop. Subsequent heat treatment depolymerized the chain structure back to the binuclear configuration, which interrupted the slow magnetic relaxation, resulting in a reversible on–off SMM behavior. Concurrently, the lattice strain generated by cycloaddition propagates anisotropically, inducing macroscopic rolling and flipping motions in the crystalline samples, and intensely bending deformation in the thin film. As a result, this work for the first time achieved the photomagnetic behavior and photomechanical response in functional molecules.

## 2 Results and Discussion

**Figure 1:**
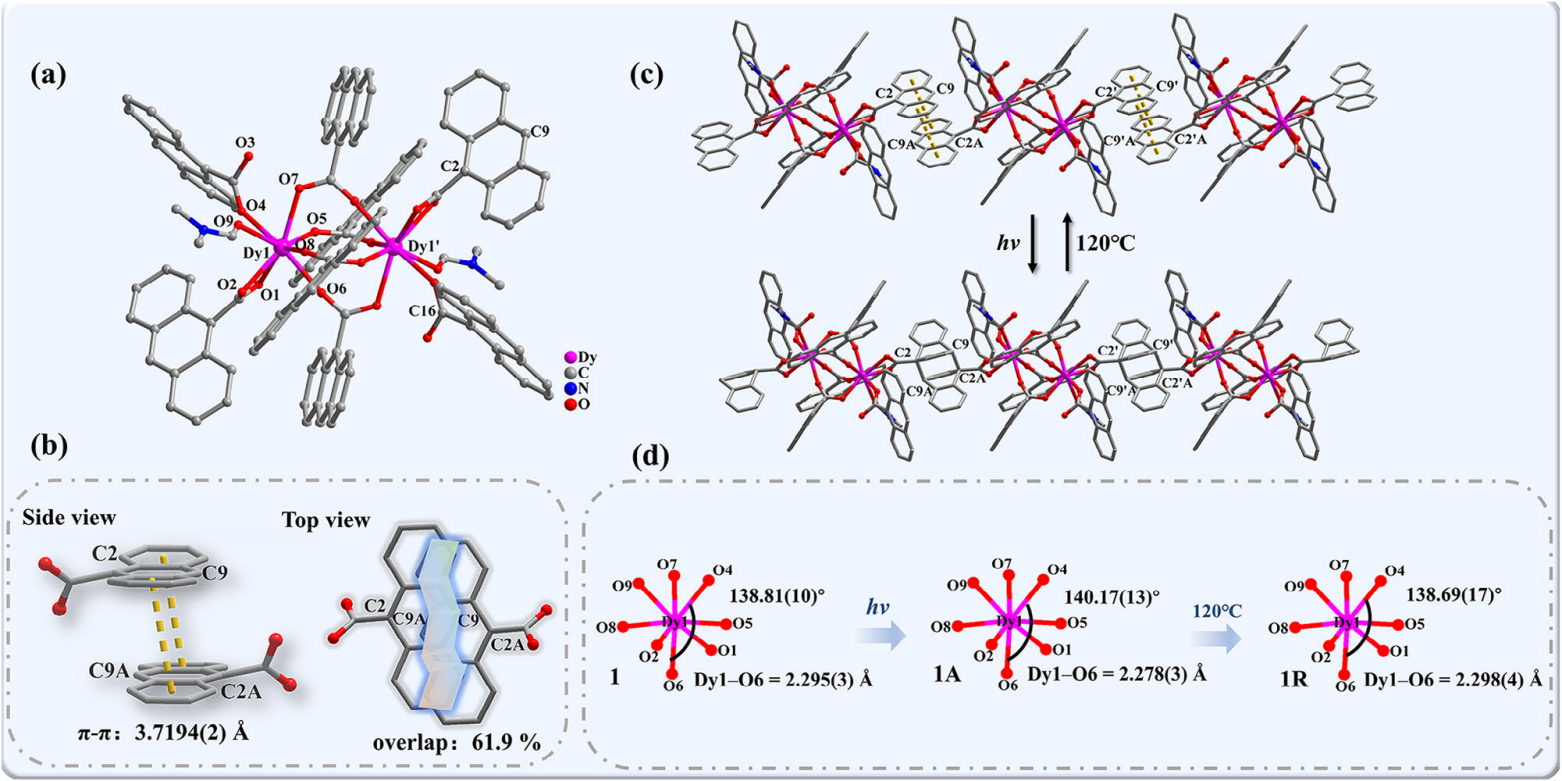
Crystal structure of **1**: a) binuclear molecular structure; b) π···π stacking interactions and overlapped area of the AC dimer; c) reversible [4 + 4] cycloaddition via light and temperature; d) coordination sphere of Dy1 for **1**, **1A**, and **1R**. H atoms are omitted for clarity. Color code: Dy, pink; C, gray 40%; N, blue; O, red.

The crystalline sample **1** was synthesized via solvothermal methods at 90 °C. A single‐crystal X‐ray diffraction (XRD) analysis demonstrated that the structure crystallized in the monoclinic *P*2_1_/*c* space group (Table S1, Supporting Information). As depicted in **Figure** **1**a, the molecular structure for **1** comprises two Dy^III^ ions, six deprotonated AC anions, two HAC ligands, and two DMF molecules. Notably, four AC anions serve as bridges between two Dy^III^ atoms, affording a binuclear structure. As evidenced by the thermogravimetric analysis (Figure S1, Supporting Information), the absence of crystallographic solvent molecules within the lattice voids indicates a remarkably high thermal stability, thus eliminating any potential interference of solvent molecules on the photoactivity. The Dy^III^ atom coordinates with eight O atoms, adopting an eight‐coordinated configuration with seven O atoms from AC ligands and one from the DMF molecule, showing a *D*_4d_ geometry with a deviation of 1.416 (Table S5, Supporting Information). The lengths of Dy–O bonds are in the range of 2.295(3)–2.436(3) Å, and the O–Dy–O angles lie between 54.72(10)° and 148.28(11)° (Table S2, Supporting Information). The in tramolecular and nearest intermolecular Dy···Dy distances are 4.3362(1) and 11.7119(4) Å, respectively. These results indicate a relatively weak uniaxial magnetic anisotropy and magnetic couplings between intramolecular Dy^III^ ions.^[^ ^48^, ^49^ ^]^ The interplanar π···π distance between the adjacent anthracene rings is 3.7194(2) Å with an intermolecular C2···C9A distance of 3.4886(1) Å, and the overlapped area is ≈61.9%, indicating a strong π···π interaction (Figure 1b).^[^ ^50^ ^]^ Therefore, in principle, the present compound is suitable for [4 + 4] photocycloaddition reaction after light irradiation.

**Figure 2:**
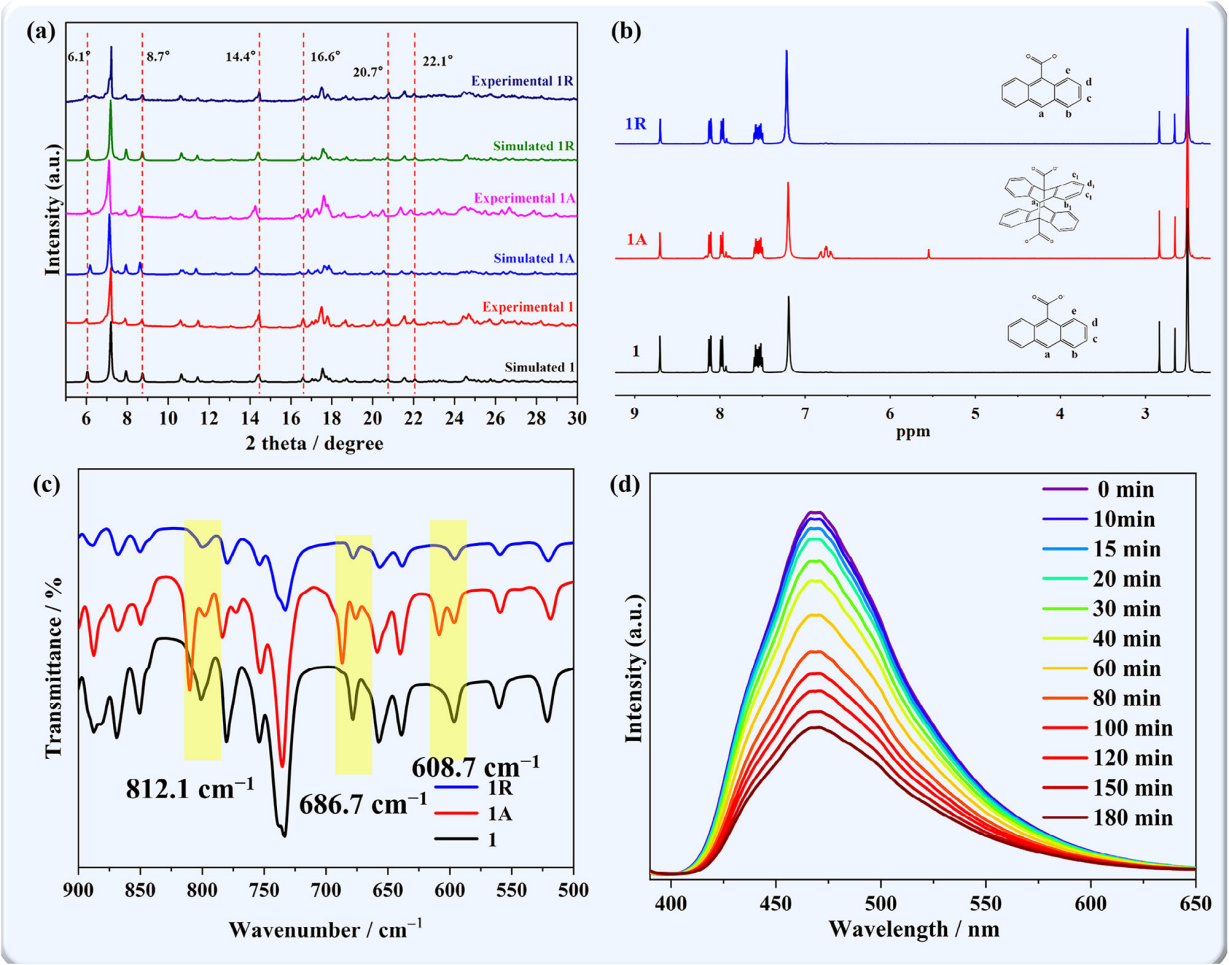
a) The PXRD plots of compounds **1**, **1A**, and **1R** b) ^1^H NMR spectra of **1**, **1A** and **1R** in 0.6 mL DMSO‐d6 and 0.5 µL DCl; c) IR spectra of **1**, **1A**, and **1R** between 900 and 500 cm^−1^ d) The in situ photoluminescence (PL) spectra of compound **1**.

Crystalline samples of the complex were subjected to powder XRD (PXRD) measurements, and the observed diffraction peaks aligned with the simulated data, which confirmed the purity of the prepared samples (**Figure** **2**a). To further investigate the occurrence of photocycloaddition reaction, the compound was subjected to ^1^H NMR and IR spectroscopy analyses. As shown in Figures 2b and S2 (Supporting Information), two signals attributable to H atoms of the dianthracene units were observed at 5.63 and 6.75 ppm. In addition, three new peaks emerged at 608.7, 686.7, and 812.1 cm^−1^, corresponding to the C–H out‐of‐plane deformation vibration of dianthracenes resulting from photocycloaddition (Figure 2c).^[^ ^51^ ^]^ The room‐temperature solid‐state PL study of **1** revealed a 465 nm emission peak (*λ*_ex_ = 360 nm), attributed to intraligand *π–π* ^*^/n–π^*^ transitions in AC components. Upon light irradiation, the fluorescence intensity decreased (Figure 2d) due to the twisty molecule conformation, which reduced the degree of molecular conjugation.^[^ ^52^, ^53^, ^54^ ^]^ Collectively, these spectral analyses provide robust support for the occurrence of photoinduced [4 + 4] of anthracene.

The photoactive behavior of **1** was convincingly demonstrated by the single‐crystal XRD data. After irradiation, the C2 and C9 atoms in the adjacent molecules formed covalent bonds, with the C2–C9A/C9–C2A distance becoming 1.642(7) Å for **1A**, respectively (Figure 1c). As a result, the binuclear structure experienced an SC‐SC transformation, evolving into a chain architecture with a [4 + 4] cycloaddition occurring between adjacent anthracene groups. Furthermore, a noteworthy reduction in the shortest Dy1–O6 bond length from 2.295(3) to 2.278(3) Å and an increase in the O6–Dy–O4 angle from 138.81(10)° to 140.17(13)° were observed (Figure 1d). These changes around the Dy center in compound **1A** are expected to enhance the uniaxial magnetic anisotropy, thereby contributing to the observed slow magnetic relaxation.^[^ ^55^ ^]^

**Figure 3:**
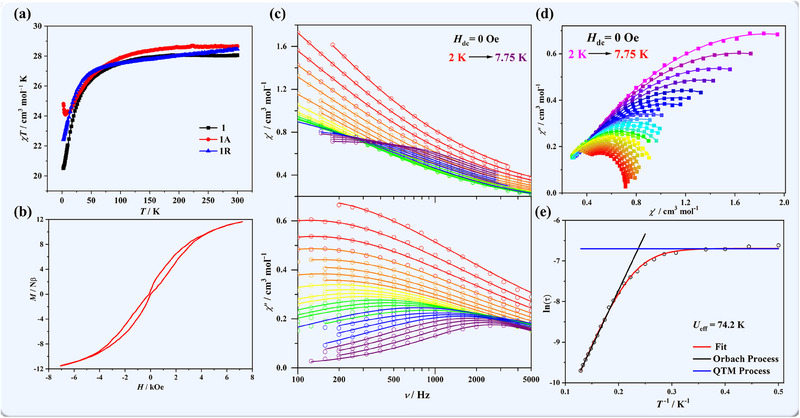
a) Variable‐temperature dc magnetic susceptibility of compounds **1**, **1A**, and **1R** under a dc field of 1000 Oe between 2 and 300 K; b) the hysteresis loops of **1A** at 1.8 K with a variable field velocity of 700 Oe s^−1^; c) the frequency dependence *χ′* and *χ′′* ac susceptibility curves of **1A** under 0 Oe dc and 5 Oe ac field between 2 and 7.75 K. Solid lines were fitted using a generalized Debye relaxation model.; d) Cole–Cole plots of compound **1A** under a 0 Oe dc field; e) ln(*τ*) versus *T* ^−1^ plots for **1A**. The red lines represent the fitting results.

Motivated by the photoactive nature of **1**, we measured the magnetic susceptibility before and after light irradiation to gain more insight into its photomagnetic behavior. Initially, variable‐temperature direct current (dc) magnetic susceptibility measurements were performed in a temperature range from 2 to 300 K under a 1000 Oe field (**Figure** **3**a). The *χT* value of **1** at 300 K was 28.05 cm^3^ mol^−1^ K, marginally below the value for two isolated Dy^III^ ions of 28.34 cm^3^ mol^−1^ K. Upon cooling, the *χT* values gradually decreased to the lowest value of 20.51 cm^3^ mol^−1^ K at 2 K, suggesting the presence of weak antiferromagnetic coupling or thermal depopulation of the excited stark levels of Dy^III^ ions. The *M–H* curve recorded at 2 K revealed a linear increase in *M* values within the low‐field range, eventually reaching 11.00 Nβ at 50 kOe (Figure S3, Supporting Information). The results of alternating current (ac) magnetic susceptibility measurements (Figure S4, Supporting Information) did not exhibit any slow magnetic relaxation above 2 K, likely due to the weak uniaxial magnetic anisotropy and magnetic couplings between Dy^III^ ions. In stark contrast, compound **1A** displayed considerable variations in its magnetic behavior. In the dc magnetic susceptibility curve (Figure 3a), the *χT* value of **1A** was 28.57 cm^3^ mol^−1^ K at room temperature, decreased gradually up to reaching a temperature of 6 K, and then it increased sharply to 24.82 cm^3^ mol^−1^ K at 2 K, indicating a ferromagnetic behavior after irradiation. The *M–H* curve for **1A** at 2 K exhibited a more rapid increase in the low‐field region, reaching 12.57 Nβ at 50 kOe (Figure S3, Supporting Information). Surprisingly, **1A** displayed a butterfly‐type hysteresis loop with a quantum tunnelling of magnetization (QTM) process near to zero field (Figure 3b; Figure S5, Supporting Information), which is rare in light‐induced molecular magnets.

Given the sensitivity of magnetic relaxation dynamics to the local environments and coupling interactions of the Dy^III^ centers, detailed ac magnetic measurements were carried out under a 0 dc and 5 Oe oscillating field. As depicted in Figures 3c and S6 (Supporting Information), both the in‐phase (*χ′*) and out‐of‐phase (*χ′′*) ac susceptibilities displayed a notable temperature and frequency dependence between 100 and 5000 Hz, indicating the presence of slow magnetic relaxation, a distinctive feature of SMMs. The low‐temperature tails observed in the curves suggest the presence of accelerated relaxation processes or QTM.^[^ ^56^ ^]^ Immediately, the general Debye relaxation model was used to fit the ac magnetic susceptibilities and the Cole–Cole curves in the temperature range of 2–7.75 K (Figure 3d). From the relaxation time (*τ*), a broad range of distribution coefficients (*α*) spanning from 0.09 to 0.52 was obtained (Table S6, Supporting Information), indicating a multiple relaxation process.^[^ ^57^ ^]^ The data aligned well with the equation *τ* ^−1^ = *τ*_0_^−1^ exp(*−U_eff_*/*k*_B_ *T*) + *τ*_QTM_^−1^. At higher temperatures, the relaxation time adhered to the Arrhenius law associated with the Orbach process, which is characterized by an effective barrier of *U_eff_* = 74.2 K and a pre‐exponential factor of *τ*_0_ = 1.53 × 10−8 s. Conversely, at lower temperatures, the relaxation process was primarily governed by QTM, with a relaxation time of *τ*_QTM_ = 1.24 × 10^−3^ s (Figure 3e).

In general, the reversible processes of photodimerization can be triggered via heat treatment. Therefore, irradiated sample **1A** was heated at 120 °C for 12 h, and the resulting sample was labeled as **1R**, respectively. A single‐crystal XRD analysis unambiguously demonstrated the completion of the dedimerization process (Figure 1c; Table S1, Supporting Information). This remarkable reversibility was closely tracked using ^1^H NMR, PXRD, and IR spectroscopic analyses (Figure 2; Figure S4, Supporting Information), which confirmed that the photocycloaddition product returned to its original binuclear configuration. Magnetic susceptibility measurements were then performed on **1R** to further investigate this enigmatic reversibility. The *χT−T* curve exhibited a similar tendency to that of **1**, without the upward shift observed at low temperatures (Figure 3a). Notably, the frequency dependence of *χ′* and *χ′′* vanished, returning to the initial state of the original compound **1** (Figure S7, Supporting Information). This observation suggests that the SMM behavior can be effectively extinguished through thermal treatment. In the structural analysis of **1R**, the shortest Dy1–O6 bond length underwent a slight elongation from 2.278(3) to 2.298(4) Å and a decrease in the O6–Dy–O4 angle from 140.17(13)° to 138.69(17)° (Figure 1d). This restoration of the bond and angle around the Dy center in **1R** subsequently weakened the uniaxial magnetic anisotropy and hindered the slow relaxation process, thereby explaining the disappearance of the SMM properties. Meanwhile, the reversibility of the cycloaddition and depolymerization process was confirmed by detecting the intensity of the IR absorption at 812 cm^−1^ via alternative xenon light irradiation and heat treatment (Figure S8, Supporting Information). Compared with the photopolymerization process, the reaction rate of pyrolysis polymerization is much slower.^[^ ^45^, ^58^ ^]^ This slow reversion kinetics probably results from the high activation energy barrier for depolymerization.

**Figure 4:**
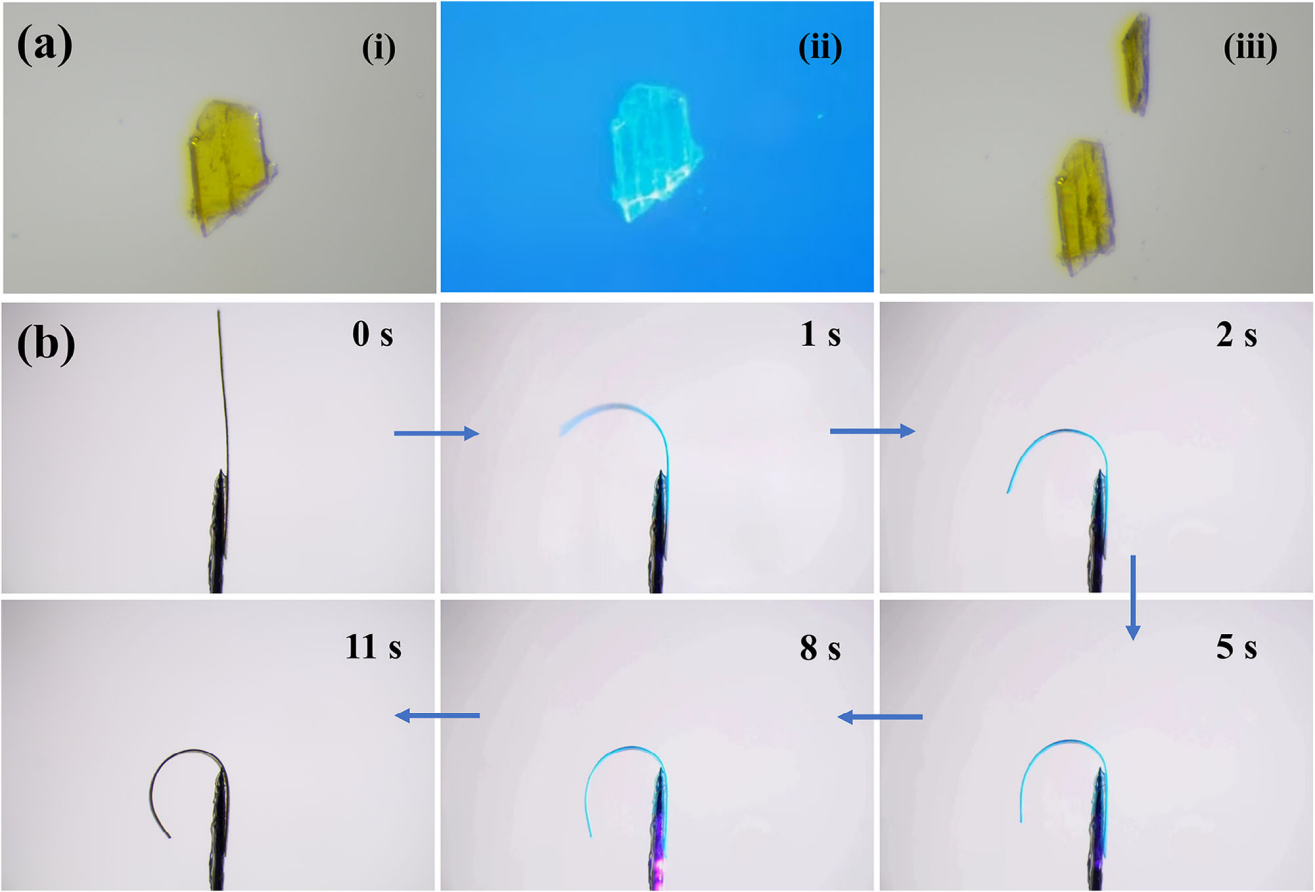
a) Cracking‐expansion features of block‐like crystals, under irradiation with 365 nm light. b) Photomechanical motions of **PVA‐1** upon exposure to light with 365 nm light.

Except for the above photomagnetic behavior, a series of photomechanical motions of single crystals were witnessed during the photodimerization triggered photopolymerization reaction. **1** exhibits distinct photomechanical responses upon 365 nm light irradiation dependent on crystal morphology: thicker specimens undergo cleavage along crystallographic planes, while thinner counterparts demonstrate complex motility including rolling and flipping (**Figure** **4**a; Videos S1 and S2, Supporting Information). This behavior originates from anisotropic lattice strain generated during the photoinduced [4+4] cycloaddition reaction, as evidenced by SC‐SC transformation analysis.^[^ ^19^ ^]^ The unit cell parameters of photoproduct **1A** revealed anisotropic expansion along the *b* (1.32%) and *c* (0.91%) axes, with a marginal volumetric increase from 4898 to 4905 Å^3^ (Δ*V* = 0.14%). The directional accumulation of internal stress from this constrained lattice reorganization induces instantaneous release during irradiation, leading to crystal fracture and motion.^[^ ^59^ ^]^

To amplify photoinduced microscopic stress into macroscopic mechanical motion, we developed a polymer composite film **PVA‐1**, through integration of a well‐ground crystalline powder sample of **1** into a polyvinyl alcohol (PVA) matrix. This composite material combines the photoresponsive properties of molecular crystals with the mechanical stability and flexibility of PVA films, yielding precise, controllable mechanical responses upon light irradiation. Powder XRD confirms retention of the crystalline phase within the composite, with distinct diffraction patterns corresponding to both **1** and PVA (Figure S9, Supporting Information). Photomechanical testing under 365 nm irradiation reveals rapid actuation, with the **PVA‐1** film achieving 90° bending deformation within 8 s (Figure 4b; Video S3, Supporting Information). This dramatic amplification effect arises from synergistic interactions between the crystalline photoresponsive component and viscoelastic polymer matrix, where localized [4+4] cycloaddition‐induced lattice strain is transmitted through hydrogen‐bonding networks to produce macroscopic motion. This crystalline‐polymer synergy enables programmable macroscopic actuation from molecular‐scale transformations while overcoming crystalline brittleness and preserving photomechanical precision, suggesting applications spanning microfluidics, soft robotics, and adaptive optics.

**Figure 5:**
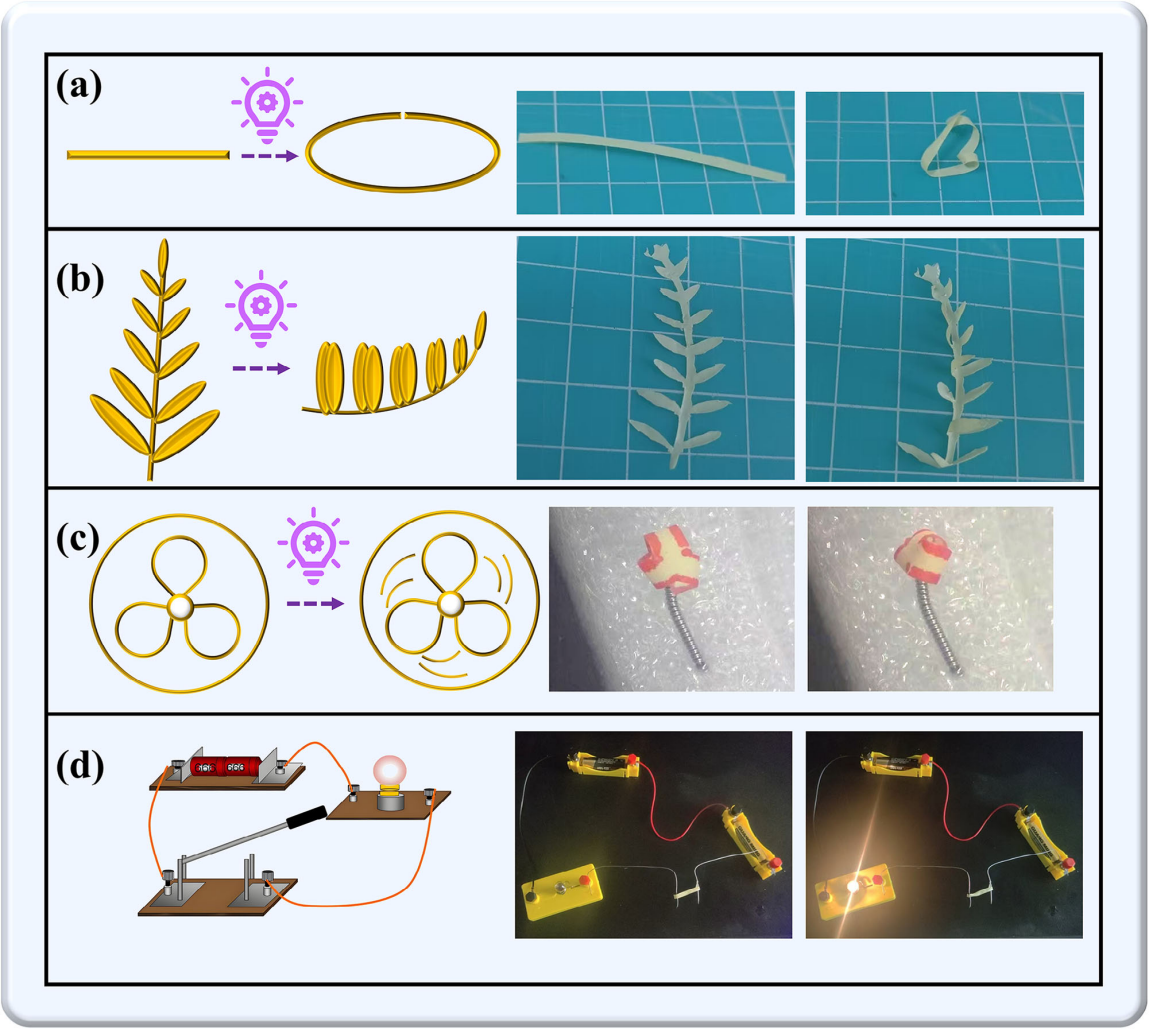
Cartoons (left) and photographic snapshots (right) of the deformations and motions of **PVA‐1**. a) curling of a **PVA‐1** onto a surface; b) leaf closure similar to a mimosa pudica; c) Imitate the rotation of a small fan; d) photoelectric switch.

The intrinsic photomechanical properties of **PVA‐1** composite films enable sophisticated light‐driven actuation with versatile device integration capabilities. We first demonstrate self‐assembly of planar architectures into closed‐loop structures through controlled photostimulation. As shown in **Figure** **5**a and Video S4 (Supporting Information), unilateral 365 nm irradiation of a rectangular **PVA‐1** film induced anisotropic lattice contraction along the illuminated edge, achieving complete loop closure within 10 s. Expanding this principle to biomimetic applications, we engineered an artificial actuator replicating the hypersensitive closure phenomenon of Mimosa pudica leaves. The **PVA‐1** film was tailored into a leaf‐shaped configuration (Figure 5b; Video S5, Supporting Information) where localized UV irradiation triggered rapid leaf closure through preferential contraction in irradiated regions. Furthermore, we developed a miniaturized rotary actuator system. A fan‐shaped **PVA‐1** structure was mounted on a needle tip within an aerodynamically isolated transparent chamber (Figure 5c). Under continuous 365 nm illumination, the photomechanical energy conversion actuated the rotational motion (Video S6, Supporting Information).

A photoswitch prototype was engineered utilizing the macroscopic photomechanical response of **PVA‐1** composite, where light‐induced film displacement achieved electrical contact closure. The validation circuit integrated a resistive load (incandescent bulb), power supply, and current‐carrying **PVA‐1** film assembly. One end of the film was fixed to a static electrode, while the other remained free, maintaining an open circuit at equilibrium. Upon light exposure, the film rapidly bent, closed the circuit, and activated the bulb (Figure 5d; Video S7, Supporting Information). The measured photomechanical displacement exceeded the operational threshold, validating the feasibility of this approach.

## 3 Conclusion

In summary, by virtue of the photocycloaddition dimerization process, the new synthesized binuclear compound **1** was transformed into a 1D chain structure via light‐induced SC‐SC transformation. This process enhances uniaxial magnetic anisotropy, yielding photoactivated SMMs with observable butterfly hysteresis. Thermal treatment reverses the dimerization, eliminating the SMM response and enabling rare reversible on‐off switching between photo‐ and thermo‐induced magnetic states. Remarkably, the photocycloaddition‐induced lattice strain propagates anisotropically, triggering macroscopic torsional dynamics (rolling, flipping etc.) in bulk crystals and pronounced flexural deformations in thin film architectures, demonstrating light‐driven actuation and multifunctional device integration. Our findings demonstrate a pioneering integration of light‐modulated magnetic dynamics and mechanical actuation, establishing a new paradigm for multifunctional materials where photomechanical and magnetic properties exhibit cooperative enhancement beyond mere coexistence. This breakthrough demonstrates molecular engineering as a viable route to reconcile rigidity‐flexibility conflicts, the dynamic interplay between spin states and lattice strain opens avenues for developing “smart” magneto‐photoactuators, adaptive spin filters, and multistimuli‐responsive memory devices.

## Conflict of Interest

The authors declare no conflict of interest.

## Author Contributions

Y.‐H.W. and S.‐K.Y. contributed equally to this work.

## Supporting information



Supporting Information

Supplemental Video 1

Supplemental Video 2

Supplemental Video 3

Supplemental Video 4

Supplemental Video 5

Supplemental Video 6

Supplemental Video 7

## Data Availability

The data that support the findings of this study are available in the supplementary material of this article.
