# Supplementary material for: Synergistic Photomagnetic and Photomechanical Dynamics in a Dysprosium‐Based Smart Molecule
Source: Adv Sci (Weinh). 2025 Jul 13;12(37):e09088. doi: 10.1002/advs.202509088 (PMC12499438; doi:10.1002/advs.202509088)
Supplement: Supplementary file 1 — Supporting Information [file ADVS-12-e09088-s003.docx]

**Supporting Information**

Synergistic Photomagnetic and Photomechanical Dynamics in a Dysprosium-Based Smart Molecule

Yu-Han Wang^†^, Shi-Kun Yan^†^, Shuai Liang, Yan-Rui Zhao, Jin Zhang, Guo-Ming Wang,* Ji-Xiang Hu*

**Contents**

**Experimental section**

Figure S1. The TG plots of compounds **1** under N_2_ atmosphere.

Figure S2. The ^1^H NMR spectrum of compound **1**.

Figure S3. Field dependence of magnetization for **1**, **1A** and **1R** at 2 K.

Figure S4. Temperature dependence of the *χ′* and *χ′′* ac susceptibilities for **1** under a 0 Oe dc and 5 Oe ac field.

Figure S5. The hysteresis loops of compound **1A** at 2 K with a variable field velocity of 200 Oe/s.

Figure S6. Temperature dependence of the *χ*′ and *χ′′* ac susceptibilities for **1A** between 2 and 12 K under 0 Oe dc and 5 Oe ac field.

Figure S7. Temperature dependence of the *χ′* and *χ′′* ac susceptibilities for **1R** under a 0 Oe dc and 5 Oe ac field.

Figure S8. The reversibility measurements of **1**. The IR absorption intensity at 812 cm^−1^ was selected to detect the reversible phenomenon via alternative xenon lamp irradiation and heating treatment.

Figure S9. The PXRD plots of compounds **1**, **PVA-1** and **PVA.**

Table S1. Crystallographic data for compounds **1**, **1A** and **1R**.

Table S2. Selected bond lengths (Å) and angles (^o^) for compound **1** at 293 K.

Table S3. Selected bond lengths (Å) and angles (^o^) for compound **1A** at 293 K.

Table S4. Selected bond lengths (Å) and angles (^o^) for compound **1R** at 293 K.

Table S5. Continuous Shape Measure (CShM) analyses of geometries for compounds **1, 1A** and **1R** by SHAPE 2.0 Software.

Table S6. Relaxation fitting parameters from Least-Squares Fitting of *χ*(*f*) data under a 0 Oe dc field of **1A**.

**Experimental Section**

Materials and methods

The organic 9-Anthracenecarboxylic acid was purchased from *Chemsoon*. All chemicals were reagent grade and used as purchased without further purification. Elemental analyses (for C, H, and N) were performed on a Perkin-Elmer 240C analyzer (Perkin-Elmer, USA). The thermogravimetric (TG) analyses were performed under a N_2_ atmosphere on a Rigaku standard TG-DTA (differential thermal analysis) analyzer. IR curves for all compounds were measured on a FT-IR spectrometer (MAGNA-560 (Nicolet)) at room temperature with KBr pellets. The time-dependent luminescence spectra were performed on a Hitachi F-4700 Fluorescence spectrometer. The UV-Vis spectrometer was recorded on a Puxi Tu-1901 spectrophotometer using BaSO_4_ as a reference. The room temperature EPR spectroscopy was recorded on a CIQTEK EPR200-Plus with continues-wave X band frequency. Direct current magnetic susceptibility measurements of the samples were recorded on a Quantum Design MPMS magnetometer. Powder X-ray diffraction (PXRD) spectra were performed through Rigaku standard MiniFlex600 and HyPix diffractometers. Simulation of the PXRD curve was carried out by the single-crystal data and diffraction-crystal module of the Mercury (Hg) program. A Perfect Light PLS-SXE 300 W Xenon lamp (320−780 nm), and a high-power UVLED spot lamp source (HEIGHT-LED, HTLD-4II) (365 nm, 405 nm, 450 nm) were equipped to prepare the photo-responsive experiments. Crystal samples were grinded to powders and the light irradiation time was elongated to 2 h to ensure the full reaction of the photo-responsive experiments.

**X-ray crystallography**

The single-crystal XRD data of **1**, **1A**, **1R**, were collected on a Rigaku XtaLAB Synergy R, DW system, HyPix diffractometer with Mo-Kα radiation. The SHELX-2016 software was used to solve the structures. Detailed crystallographic data for **1**, **1A** and **1R** were summarized in Tables S1 and S2, and the selected bond lengths and angles were listed in Tables S3−S8. Full crystallographic data for all compounds have been deposited with the CCDC numbers of 2382957 for **1**, 2382958 for **1A**, 2382959 for **1R**.

**Synthesis of [Dy_2_(DMF)_2_(HAC)_2_(AC)_6_] (1):**

A mixture of DyCl_3_·6H_2_O (0.037 g, 0.098 mmol) and 9-HAC (0.03 g, 0.135 mmol) was dissolved in a solvent blend comprising 1 mL DMF and 5 mL H_2_O, within a 20 mL glass vial. This solution was subsequently heated to 90 °C for a duration of 12 h, followed by a gradual cooling process down to 30 °C, yielding the crystalline product. Yield: ca.50% based on 9-HAC ligands. Elemental analysis for **1** (%): calcd for C_126_H_88_Dy_2_N_2_O_18_ (2242.98): C, 67.47; H, 3.95; N, 1.25. Found: C, 67.38; H, 3.88; N, 1.32. IR of **1** (KBr pellets, cm^−1^): 3419(m), 3032(w), 2911(w), 2836(w), 2359(w), 1609(s), 1519(m), 1375(s), 1306(w), 1253(m), 1102(w), 1003(w), 887(w), 868(s), 849(w), 800(s), 780(s), 755(s), 734(s), 677(w), 656(w), 638(m), 595(w), 559(w), 521(w).

**Synthesis of [Dy_2_(DMF)_2_(HAC)_2_(AC)_6_]_n_ (1A):**

Compound **1A** was obtained by Xe-lamp irradiation of compound **1** at room temperature for 10 min. Elemental analysis for **1A** (%): calcd for C_126_H_88_Dy_2_N_2_O_18_ (2242.98): C, 67.47; H, 3.95; N, 1.25. Found: C, 67.50; H, 3.90; N, 1.29. IR of **1A** (KBr pellets, cm^−1^): 3411(m), 3040(w), 2911(w), 2821(w), 2351(w), 1601(s), 1526(m), 1375(s), 1306(w), 1253(m), 1102(w), 996(w), 888(w), 867(w), 849(w), 810(w), 796(w), 784(w), 772(w), 753(w), 736(s), 687(w), 675(w), 658(w), 639(m), 608(w), 595(w), 559(w), 518(w).

**Synthesis of [Dy_2_(DMF)_2_(HAC)_2_(AC)_6_] (1R):**

Compound **1R** was obtained via thermal treatment of compound **1A** at 120 ℃ for 12 h. Elemental analysis for **1R** (%): calcd for C_126_H_88_Dy_2_N_2_O_18_ (2242.98): C, 67.47; H, 3.95; N, 1.25. Found: C, 67.55; H, 4.01; N, 1.19. IR of **1R** (KBr pellets, cm^−1^): 3438(m), 3038(w), 2927(w), 2839(w), 1619(s), 1529(m), 1435(s), 1388(w), 1311(w), 1259(m), 1106(w), 1012(w), 887(w), 866(m), 849(m), 798(m), 780(w), 753(m), 731(s), 678(m), 657(m), 637(m), 596(w), 558(w), 521(w).

**Preparation of the Composite Membrane of 1**-**PVA:**

The composite membrane **1-PVA** were fabricated by a mixture of **1** and 3 wt% water solutions of polyvinyl alcohol (PVA). The polycrystalline particle of **1** were ground into a fine powder. Afterwards, 3 g PVA solution (3 wt%) was mixed with **1** (50 mg) and stirred overnight at room temperature for a uniform solution. The uniform solution was then directly drop cast onto smooth and clean glass pane (25.4 × 76.2 mm^2^) and dried under room temperature overnight to remove any residual solvents. After the solvents were completely evaporated, the freestanding membrane thickness was obtained by peeling it off from the glass pane.


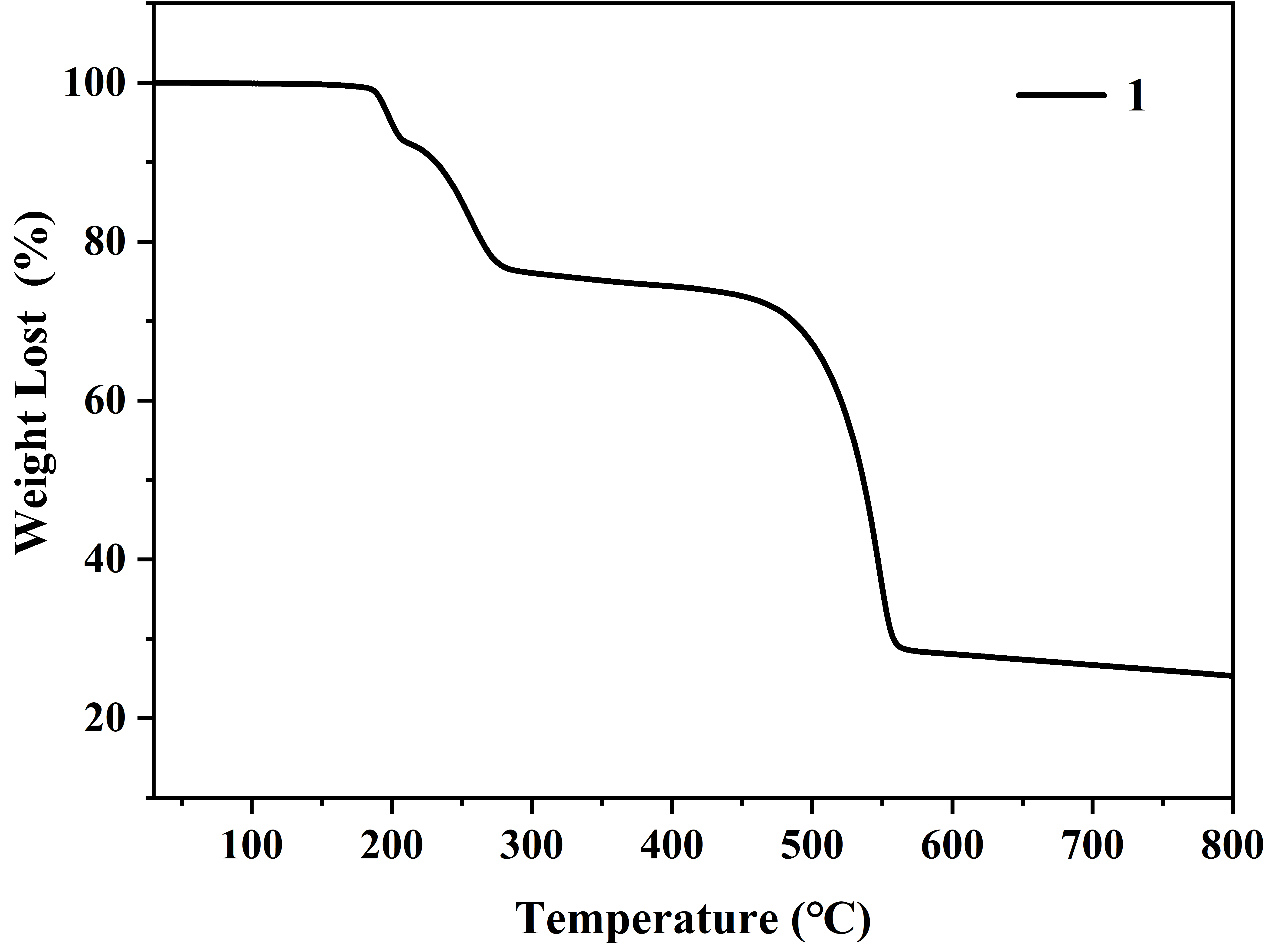


Figure S1. The TG plots of compounds **1** under N_2_ atmosphere.


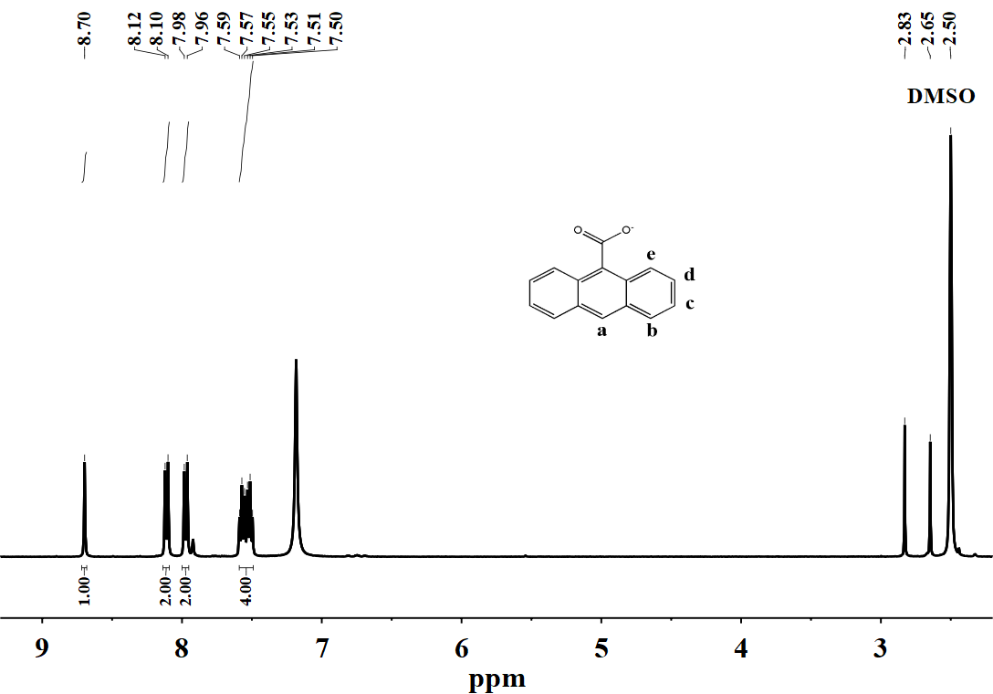


Figure S2. The ^1^H NMR spectrum of compound **1**.


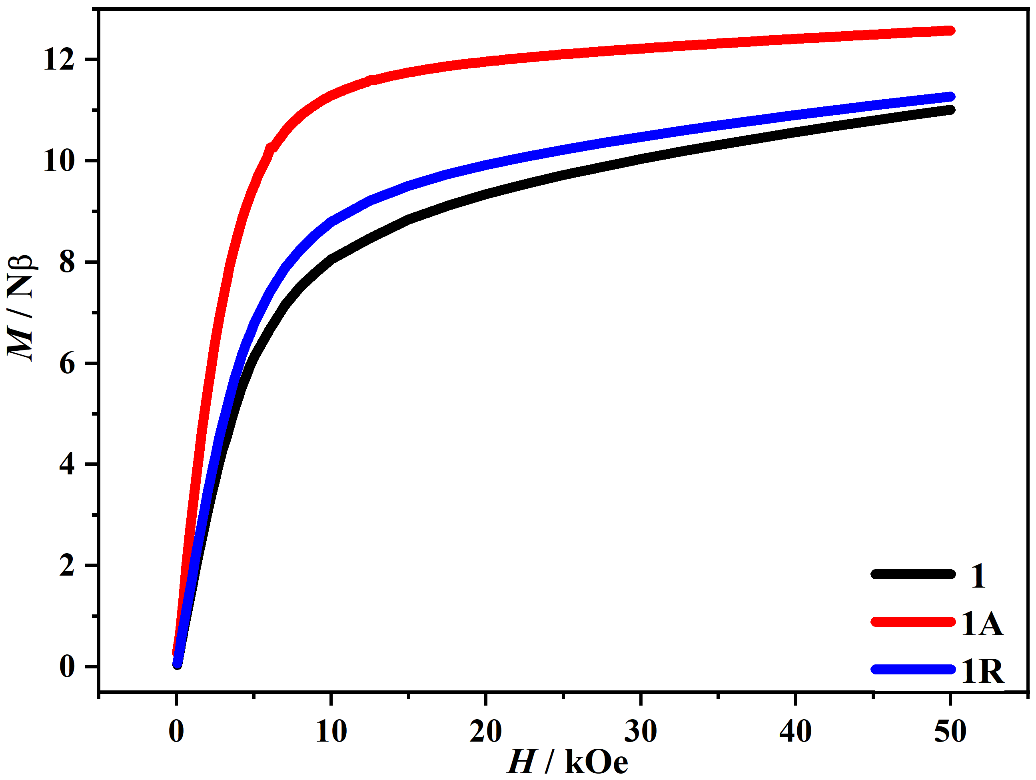


Figure S3. Field dependence of magnetization for **1**, **1A** and **1R** at 2 K.





Figure S4. Temperature dependence of the *χ′* and *χ′′* ac susceptibilities for **1** under a 0 Oe dc and 5 Oe ac field.


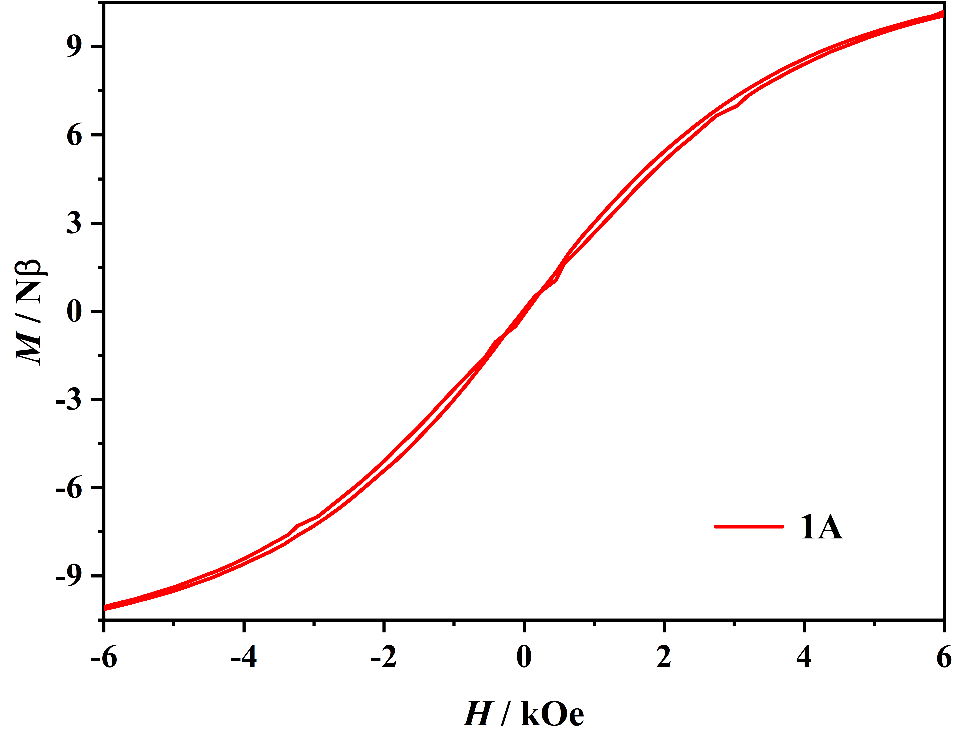


Figure S5. The hysteresis loops of compound **1A** at 2 K with a variable field velocity of 200 Oe/s.


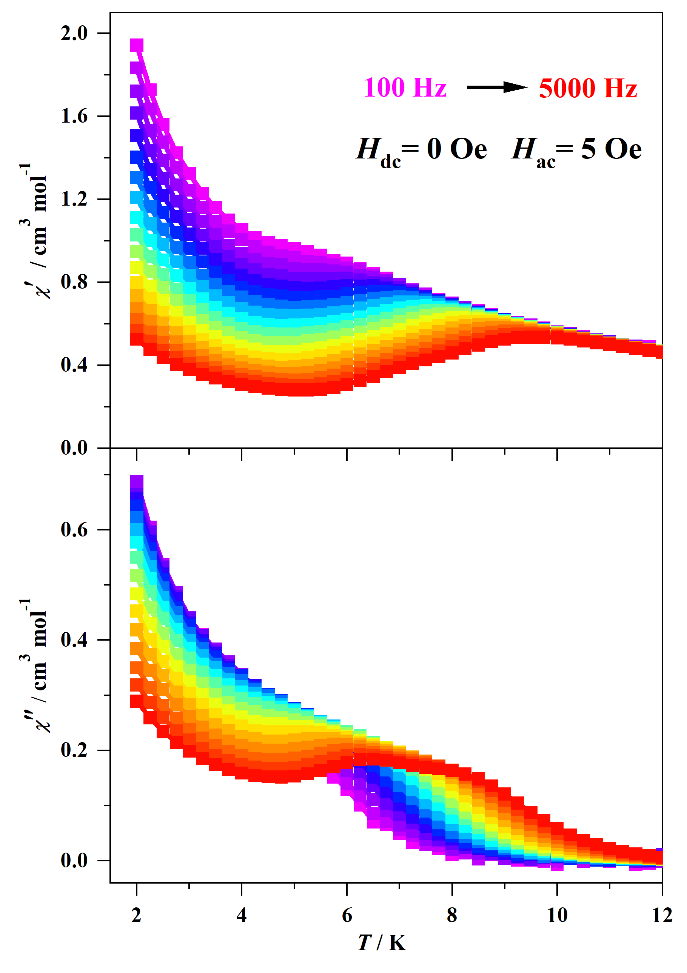


Figure S6. Temperature dependence of the *χ*′ and *χ′′* ac susceptibilities for **1A** between 2 and 12 K under 0 Oe dc and 5 Oe ac field.


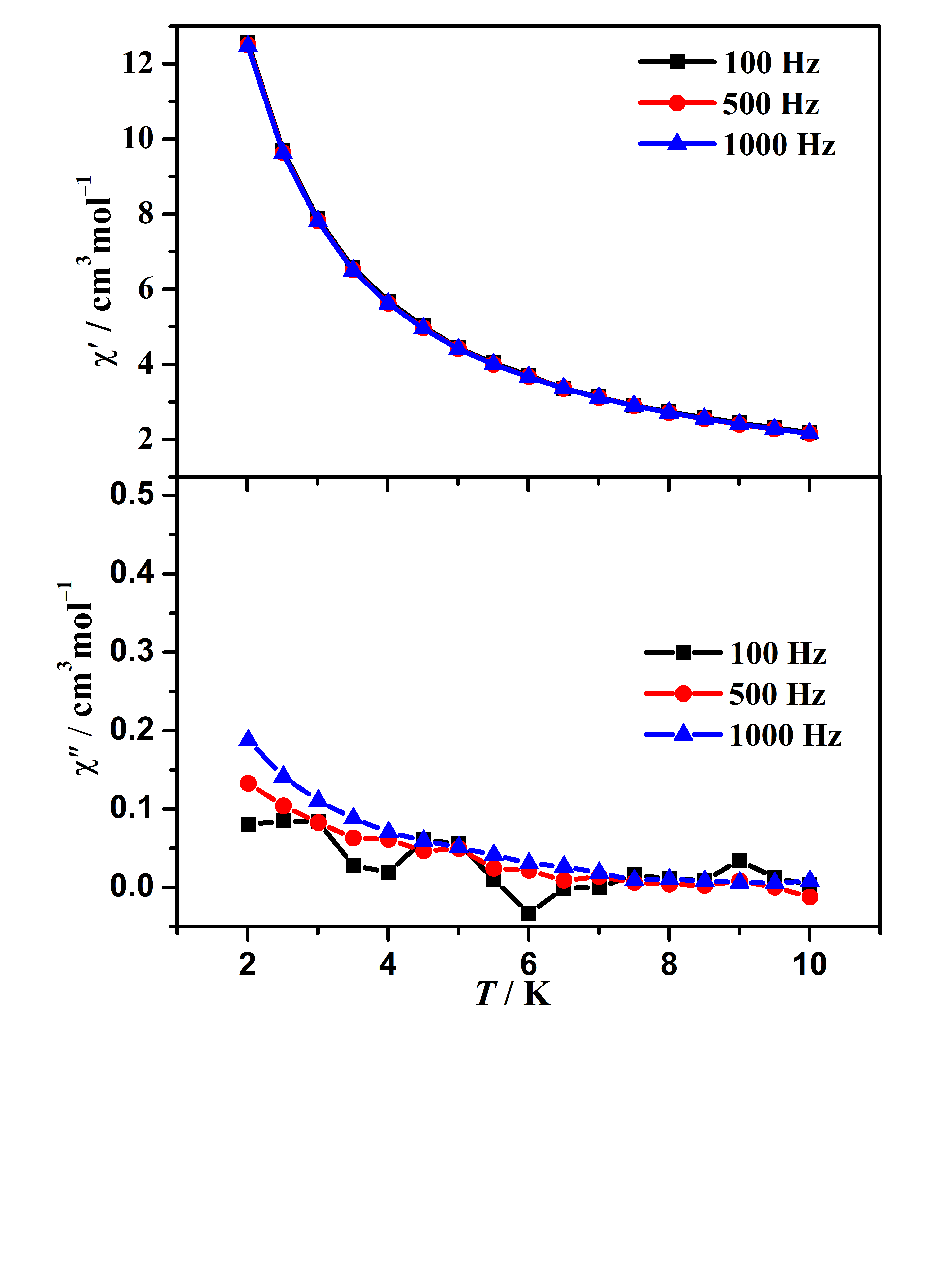


Figure S7. Temperature dependence of the *χ′* and *χ′′* ac susceptibilities for **1R** under a 0 Oe dc and 5 Oe ac field.


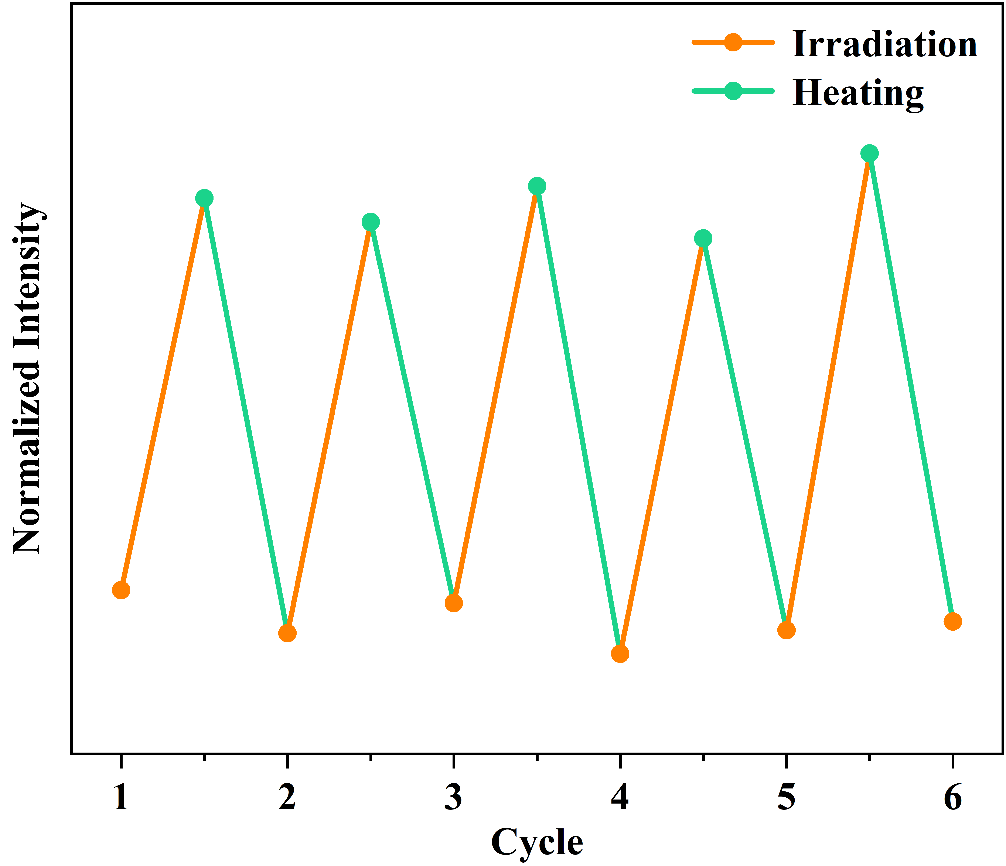


Figure S8. The reversibility measurements of **1**. The IR absorption intensity at 812 cm^−1^ was selected to detect the reversible phenomenon via alternative xenon lamp irradiation and heating treatment.


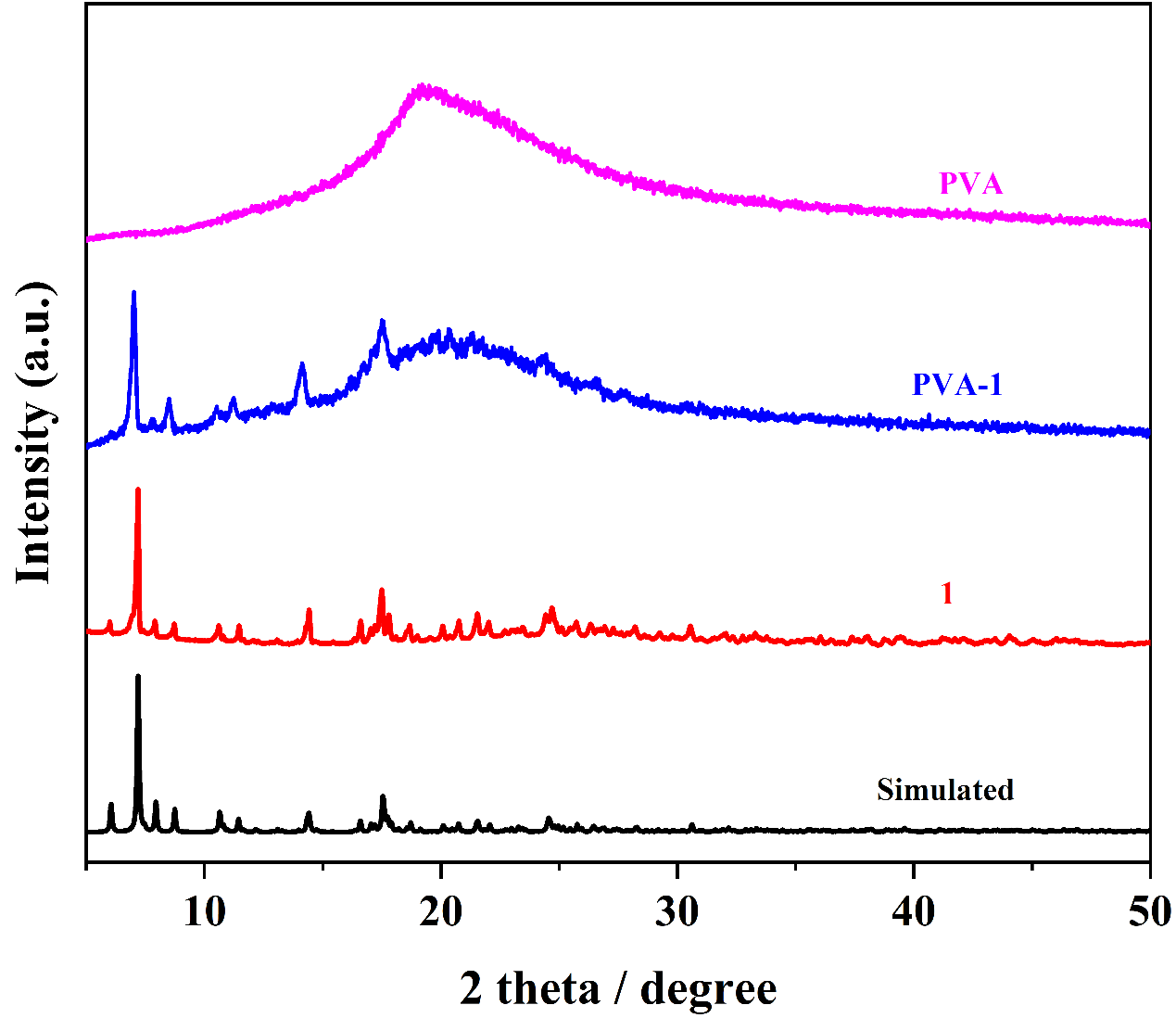


Figure S9. The PXRD plots of compounds **1**, **PVA-1** and **PVA.**

Table S1. Crystallographic data for compounds **1**, **1A** and **1R**.

|  | **1** | **1A** | **1R** |
| --- | --- | --- | --- |
| Formula | C_126_H_88_Dy_2_N_2_O_18_ | C_126_H_88_Dy_2_N_2_O_18_ | C_126_H_88_Dy_2_N_2_O_18_ |
| *M*r (g·mol^-1^) | 2242.98 | 2242.98 | 2242.98 |
| Temperature | 293(2) K | 293(2) K | 293(2) K |
| Space group | *P*2_1_/*c* | *P*2_1_/*c* | *P*2_1_/*c* |
| Crystal system | Monoclinic | Monoclinic | Monoclinic |
| *a* (Å) | 15.6762(5) | 15.3879(6) | 15.6344(6) |
| *b* (Å) | 20.2124(6) | 20.4791(7) | 20.2307(7) |
| *c* (Å) | 16.6108(7) | 16.7617(6) | 16.6302(7) |
| *α* (*°*) | 90 | 90 | 90 |
| *β* (*°*) | 111.443(4) | 111.780(4) | 111.404(4) |
| γ (*°*) | 90 | 90 | 90 |
| *V* (Å^3^) | 4898.9(3) | 4905.1(3) | 4897.3(3) |
| *Z* | 2 | 2 | 2 |
| *F* (000) | 2268 | 2268 | 2268 |
| *Dc* (gcm^-3^) | 1.521 | 1.519 | 1.521 |
| *μ* (mm^-1^) | 1.589 | 1.587 | 1.590 |
| *R*_int_ | 0.0737 | 0.0546 | 0.0791 |
| limiting indices | -18<=h<=18  -24<=k<=24  -19<=l<=19 | -18<=h<=18  -24<=k<=24  -19<=l<=19 | -18<=h<=18  -24<=k<=24  -19<=l<=19 |
| Collected reflections | 46654 | 42796 | 41375 |
| Unique reflections | 8622 | 8647 | 8628 |
| GOF on *F*^2^ | 1.017 | 1.041 | 1.087 |
| *R*_1_, *wR*_2_ [*I*>2*σ*(*I*)] | 0.0344 0.0713 | 0.0435 0.1010 | 0.0525 0.1243 |
| *R*_1_, *wR*_2_ [all data] | 0.0527 0.0792 | 0.0555 0.1080 | 0.0812 0.1508 |
| *^a^R*_1_ =∑ \|\|*F*_0_\| − \|*F*_c_\|\|/∑\|*F*_o_\|. *^b^*w*R*_2_ = {∑ [*w*(*F*_0_^2^ − *F*_c_^2^)^2^]/∑*w*(*F*_0_^2^)^2^}^1/2^. | | | |

Table S2. Selected bond lengths (Å) and angles (^o^) for compound **1** at 293 K.

| **1** | | | |
| --- | --- | --- | --- |
| Dy(1)-O(6) | 2.295(3) | C(18)-C(23) | 1.431(6) |
| Dy(1)-O(7)#1 | 2.323(3) | C(19)-C(20) | 1.372(7) |
| Dy(1)-O(5) | 2.328(3) | C(20)-C(21) | 1.402(9) |
| Dy(1)-O(2) | 2.354(3) | C(21)-C(22) | 1.329(9) |
| Dy(1)-O(9) | 2.404(3) | C(22)-C(23) | 1.417(8) |
| Dy(1)-O(8) | 2.406(3) | C(23)-C(24) | 1.380(8) |
| Dy(1)-O(1) | 2.426(3) | C(24)-C(25) | 1.379(8) |
| Dy(1)-O(4) | 2.436(3) | C(25)-C(26) | 1.417(8) |
| C(16)-O(4) | 1.215(5) | C(25)-C(30) | 1.433(7) |
| C(16)-O(3) | 1.311(5) | C(26)-C(27) | 1.339(9) |
| C(16)-C(17) | 1.490(6) | C(27)-C(28) | 1.397(9) |
| C(17)-C(30) | 1.396(6) | C(28)-C(29) | 1.358(7) |
| C(17)-C(18) | 1.412(6) | C(29)-C(30) | 1.428(7) |
| C(18)-C(19) | 1.419(7) |  |  |
|  |  |  |  |
| O(6)-Dy(1)-O(7)#1 | 73.62(10) | O(4)-C(16)-O(3) | 123.3(4) |
| O(6)-Dy(1)-O(5) | 123.34(9) | O(4)-C(16)-C(17) | 122.6(4) |
| O(7)#1-Dy(1)-O(5) | 80.24(10) | O(3)-C(16)-C(17) | 114.1(4) |
| O(6)-Dy(1)-O(2) | 93.60(10) | C(30)-C(17)-C(18) | 121.1(4) |
| O(7)#1-Dy(1)-O(2) | 82.73(11) | C(30)-C(17)-C(16) | 118.3(4) |
| O(5)-Dy(1)-O(2) | 131.94(10) | C(18)-C(17)-C(16) | 120.4(4) |
| O(6)-Dy(1)-O(9) | 74.13(10) | C(17)-C(18)-C(19) | 123.5(4) |
| O(7)#1-Dy(1)-O(9) | 137.92(11) | C(17)-C(18)-C(23) | 118.7(5) |
| O(5)-Dy(1)-O(9) | 141.01(11) | C(19)-C(18)-C(23) | 117.8(5) |
| O(2)-Dy(1)-O(9) | 73.08(12) | C(20)-C(19)-C(18) | 120.2(5) |
| O(6)-Dy(1)-O(8) | 78.49(10) | C(19)-C(20)-C(21) | 120.9(6) |
| O(7)#1-Dy(1)-O(8) | 122.97(10) | C(22)-C(21)-C(20) | 120.7(6) |
| O(5)-Dy(1)-O(8) | 75.24(10) | C(21)-C(22)-C(23) | 121.2(6) |
| O(2)-Dy(1)-O(8) | 148.28(11) | C(24)-C(23)-C(22) | 121.6(5) |
| O(9)-Dy(1)-O(8) | 75.21(10) | C(24)-C(23)-C(18) | 119.1(5) |
| O(6)-Dy(1)-O(1) | 139.51(10) | C(22)-C(23)-C(18) | 119.2(6) |
| O(7)#1-Dy(1)-O(1) | 77.48(11) | C(25)-C(24)-C(23) | 122.8(5) |
| O(5)-Dy(1)-O(1) | 77.76(10) | C(24)-C(25)-C(26) | 121.2(6) |
| O(2)-Dy(1)-O(1) | 54.72(10) | C(24)-C(25)-C(30) | 119.0(5) |
| O(9)-Dy(1)-O(1) | 112.61(11) | C(26)-C(25)-C(30) | 119.8(6) |
| O(8)-Dy(1)-O(1) | 141.84(10) | C(27)-C(26)-C(25) | 120.0(6) |
| O(6)-Dy(1)-O(4) | 138.81(10) | C(26)-C(27)-C(28) | 121.4(6) |
| O(7)#1-Dy(1)-O(4) | 147.54(10) | C(29)-C(28)-C(27) | 121.0(7) |
| O(5)-Dy(1)-O(4) | 78.13(10) | C(28)-C(29)-C(30) | 120.3(6) |
| O(2)-Dy(1)-O(4) | 94.06(11) | C(17)-C(30)-C(29) | 123.2(4) |
| O(9)-Dy(1)-O(4) | 69.64(10) | C(17)-C(30)-C(25) | 119.2(5) |
| O(8)-Dy(1)-O(4) | 73.96(10) | C(29)-C(30)-C(25) | 117.5(5) |
| O(1)-Dy(1)-O(4) | 74.49(10) |  |  |
| Symmetry transformations used to generate equivalent atoms: #1 -x+1, -y+2, -z | | | |

Table S3. Selected bond lengths (Å) and angles (^o^) for compound **1A** at 293 K.

| **1A** | | | | |
| --- | --- | --- | --- | --- |
| Dy(1)-O(6)#1 | 2.278(4) | | C(18)-C(23) | 1.443(10) |
| Dy(1)-O(2) | 2.321(3) | | C(19)-C(20) | 1.383(12) |
| Dy(1)-O(5) | 2.327(4) | | C(20)-C(21) | 1.437(12) |
| Dy(1)-O(7) | 2.331(4) | | C(21)-C(22) | 1.228(13) |
| Dy(1)-O(8) | 2.399(3) | | C(22)-C(23) | 1.426(13) |
| Dy(1)-O(4) | 2.407(4) | | C(23)-C(24) | 1.377(12) |
| Dy(1)-O(9) | 2.416(4) | | C(24)-C(25) | 1.315(13) |
| Dy(1)-O(1) | 2.457(3) | | C(25)-C(30) | 1.455(11) |
| C(16)-O(4) | 1.213(7) | | C(25)-C(26) | 1.465(13) |
| C(16)-O(3) | 1.315(7) | | C(26)-C(27) | 1.271(14) |
| C(16)-C(17) | 1.484(9) | | C(27)-C(28) | 1.397(14) |
| C(17)-C(18) | 1.399(10) | | C(28)-C(29) | 1.362(11) |
| C(17)-C(30) | 1.414(11) | | C(29)-C(30) | 1.409(12) |
| C(18)-C(19) | 1.401(11) | |  |  |
|  |  | |  |  |
| O(6)#1-Dy(1)-O(2) | | 91.62(13) | O(6)-Dy(1)-O(7)#1 | 73.62(10) |
| O(6)#1-Dy(1)-O(5) | | 123.69(12) | O(6)-Dy(1)-O(5) | 123.34(9) |
| O(2)-Dy(1)-O(5) | | 135.44(12) | O(7)#1-Dy(1)-O(5) | 80.24(10) |
| O(6)#1-Dy(1)-O(7) | | 75.22(14) | O(6)-Dy(1)-O(2) | 93.60(10) |
| O(2)-Dy(1)-O(7) | | 84.20(14) | O(7)#1-Dy(1)-O(2) | 82.73(11) |
| O(5)-Dy(1)-O(7) | | 80.44(14) | O(5)-Dy(1)-O(2) | 131.94(10) |
| O(6)#1-Dy(1)-O(8) | | 78.68(13) | O(6)-Dy(1)-O(9) | 74.13(10) |
| O(2)-Dy(1)-O(8) | | 144.51(13) | O(7)#1-Dy(1)-O(9) | 137.92(11) |
| O(5)-Dy(1)-O(8) | | 75.07(13) | O(5)-Dy(1)-O(9) | 141.01(11) |
| O(7)-Dy(1)-O(8) | | 124.74(12) | O(2)-Dy(1)-O(9) | 73.08(12) |
| O(6)#1-Dy(1)-O(4) | | 140.17(13) | O(6)-Dy(1)-O(8) | 78.49(10) |
| O(2)-Dy(1)-O(4) | | 92.83(13) | O(7)#1-Dy(1)-O(8) | 122.97(10) |
| O(5)-Dy(1)-O(4) | | 77.19(13) | O(5)-Dy(1)-O(8) | 75.24(10) |
| O(7)-Dy(1)-O(4) | | 144.61(13) | O(2)-Dy(1)-O(8) | 148.28(11) |
| O(8)-Dy(1)-O(4) | | 74.96(12) | O(9)-Dy(1)-O(8) | 75.21(10) |
| O(6)#1-Dy(1)-O(9) | | 74.32(13) | O(6)-Dy(1)-O(1) | 139.51(10) |
| O(2)-Dy(1)-O(9) | | 70.93(14) | O(7)#1-Dy(1)-O(1) | 77.48(11) |
| O(5)-Dy(1)-O(9) | | 139.17(14) | O(5)-Dy(1)-O(1) | 77.76(10) |
| O(7)-Dy(1)-O(9) | | 139.66(15) | O(2)-Dy(1)-O(1) | 54.72(10) |
| O(8)-Dy(1)-O(9) | | 73.59(13) | O(9)-Dy(1)-O(1) | 112.61(11) |
| O(4)-Dy(1)-O(9) | | 69.92(13) | O(8)-Dy(1)-O(1) | 141.84(10) |
| O(6)#1-Dy(1)-O(1) | | 138.13(14) | O(6)-Dy(1)-O(4) | 138.81(10) |
| O(2)-Dy(1)-O(1) | | 54.20(12) | O(7)#1-Dy(1)-O(4) | 147.54(10) |
| O(5)-Dy(1)-O(1) | | 81.63(12) | O(5)-Dy(1)-O(4) | 78.13(10) |
| O(7)-Dy(1)-O(1) | | 77.65(13) | O(2)-Dy(1)-O(4) | 94.06(11) |
| O(8)-Dy(1)-O(1) | | 143.15(13) | O(9)-Dy(1)-O(4) | 69.64(10) |
| O(4)-Dy(1)-O(1) | | 72.26(13) | O(8)-Dy(1)-O(4) | 73.96(10) |
| O(9)-Dy(1)-O(1) | | 109.55(12) | O(1)-Dy(1)-O(4) | 74.49(10) |
| Symmetry transformations used to generate equivalent atoms: #1 -x+1, -y+2, -z; #2 -x+2, -y+2, -z | | | | |

Table S4. Selected bond lengths (Å) and angles (^o^) for compound **1R** at 293 K

| **1R** | | | |
| --- | --- | --- | --- |
| Dy(1)-O(6) | 2.298(4) | C(18)-C(23) | 1.454(10) |
| Dy(1)-O(5) | 2.317(5) | C(19)-C(20) | 1.354(12) |
| Dy(1)-O(7)#1 | 2.325(5) | C(20)-C(21) | 1.415(15) |
| Dy(1)-O(2) | 2.346(5) | C(21)-C(22) | 1.328(15) |
| Dy(1)-O(9) | 2.398(5) | C(22)-C(23) | 1.415(13) |
| Dy(1)-O(8) | 2.409(4) | C(23)-C(24) | 1.382(13) |
| Dy(1)-O(4) | 2.424(4) | C(24)-C(25) | 1.376(13) |
| Dy(1)-O(1) | 2.433(5) | C(25)-C(26) | 1.403(13) |
| C(16)-O(4) | 1.217(8) | C(25)-C(30) | 1.458(11) |
| C(16)-O(3) | 1.333(8) | C(26)-C(27) | 1.331(16) |
| C(16)-C(17) | 1.499(9) | C(27)-C(28) | 1.414(16) |
| C(17)-C(18) | 1.388(10) | C(28)-C(29) | 1.349(12) |
| C(17)-C(30) | 1.406(10) | C(29)-C(30) | 1.404(12) |
| C(18)-C(19) | 1.414(11) |  |  |
|  |  |  |  |
| O(6)-Dy(1)-O(5) | 123.53(17) | O(4)-C(16)-O(3) | 123.1(6) |
| O(6)-Dy(1)-O(7)#1 | 73.61(17) | O(4)-C(16)-C(17) | 123.9(6) |
| O(5)-Dy(1)-O(7)#1 | 80.49(17) | O(3)-C(16)-C(17) | 113.0(6) |
| O(6)-Dy(1)-O(2) | 93.17(17) | C(18)-C(17)-C(30) | 122.7(7) |
| O(5)-Dy(1)-O(2) | 132.28(17) | C(18)-C(17)-C(16) | 120.9(6) |
| O(7)#1-Dy(1)-O(2) | 82.62(19) | C(30)-C(17)-C(16) | 116.3(7) |
| O(6)-Dy(1)-O(9) | 73.99(17) | C(17)-C(18)-C(19) | 124.7(7) |
| O(5)-Dy(1)-O(9) | 140.70(18) | C(17)-C(18)-C(23) | 118.6(7) |
| O(7)#1-Dy(1)-O(9) | 137.93(18) | C(19)-C(18)-C(23) | 116.7(8) |
| O(2)-Dy(1)-O(9) | 73.1(2) | C(20)-C(19)-C(18) | 121.9(9) |
| O(6)-Dy(1)-O(8) | 78.34(17) | C(19)-C(20)-C(21) | 120.2(10) |
| O(5)-Dy(1)-O(8) | 75.35(16) | C(22)-C(21)-C(20) | 120.9(11) |
| O(7)#1-Dy(1)-O(8) | 123.00(16) | C(21)-C(22)-C(23) | 121.1(10) |
| O(2)-Dy(1)-O(8) | 147.99(19) | C(24)-C(23)-C(22) | 122.1(9) |
| O(9)-Dy(1)-O(8) | 74.84(17) | C(24)-C(23)-C(18) | 118.6(8) |
| O(6)-Dy(1)-O(4) | 138.69(17) | C(22)-C(23)-C(18) | 119.2(9) |
| O(5)-Dy(1)-O(4) | 78.12(16) | C(25)-C(24)-C(23) | 123.2(8) |
| O(7)#1-Dy(1)-O(4) | 147.69(17) | C(24)-C(25)-C(26) | 121.4(9) |
| O(2)-Dy(1)-O(4) | 94.18(18) | C(24)-C(25)-C(30) | 119.2(8) |
| O(9)-Dy(1)-O(4) | 69.49(17) | C(26)-C(25)-C(30) | 119.3(10) |
| O(8)-Dy(1)-O(4) | 74.11(15) | C(27)-C(26)-C(25) | 120.8(11) |
| O(6)-Dy(1)-O(1) | 139.45(17) | C(26)-C(27)-C(28) | 120.7(10) |
| O(5)-Dy(1)-O(1) | 78.12(17) | C(29)-C(28)-C(27) | 121.0(11) |
| O(7)#1-Dy(1)-O(1) | 77.78(18) | C(28)-C(29)-C(30) | 121.0(9) |
| O(2)-Dy(1)-O(1) | 54.74(18) | C(29)-C(30)-C(17) | 125.0(7) |
| O(9)-Dy(1)-O(1) | 112.38(18) | C(29)-C(30)-C(25) | 117.2(8) |
| O(8)-Dy(1)-O(1) | 142.11(17) | C(17)-C(30)-C(25) | 117.6(8) |
| O(4)-Dy(1)-O(1) | 74.32(17) |  |  |
| Symmetry transformations used to generate equivalent atoms: #1 -x+1, -y+2, -z | | | |

Table S5. Continuous Shape Measure (CShM) analyses of geometries for compounds **1, 1A** and **1R** by SHAPE 2.0 Software.

| Geometry | **1** | **1A** | **1R** |
| --- | --- | --- | --- |
| Octagon (*D_8h_*) | 31.129 | 31.435 | 31.087 |
| Heptagonal pyramid (*C_7v_*) | 22.432 | 22.523 | 22.534 |
| Hexagonal bipyramid (*D_6h_*) | 15.058 | 16.224 | 15.164 |
| Cube (*O_h_*) | 9.491 | 10.580 | 9.559 |
| Square antiprism (*D_4d_*) | 1.416 | 1.370 | 1.395 |
| Triangular dodecahedron (*D_2d_*) | 2.244 | 2.530 | 2.279 |
| Johnson gyrobifastigium J26 (*D_2d_*) | 14.463 | 14.755 | 14.569 |
| Johnson elongated triangular bipyramid J14 (*D_3h_*) | 26.353 | 26.712 | 26.311 |
| Biaugmented trigonal prism J50 (*C_2v_*) | 2.301 | 2.206 | 2.276 |
| Biaugmented trigonal prism (*C_2v_*) | 1.422 | 1.372 | 1.412 |
| Snub diphenoid J84 (*D_2d_*) | 4.825 | 4.775 | 4.803 |
| Triakis tetrahedron (*T_d_*) | 10.281 | 11.232 | 10.319 |
| Elongated trigonal bipyramid (*D_3h_*) | 21.876 | 22.681 | 21.940 |

Table S6. Relaxation fitting parameters from Least-Squares Fitting of *χ*(*f*) data under a 0 Oe dc field of **1A**.

| *T* (K) | *χ*_T_ | *χ*_S_ | *α* | *τ*’ (s) | *τ*’’(s) |
| --- | --- | --- | --- | --- | --- |
| 2 | 3.57986 | 0.13794 | 0.51735 | 0.00135 | 0.00134 |
| 2.25 | 3.13847 | 0.13014 | 0.51503 | 0.00128 | 0.0013 |
| 2.5 | 2.78181 | 0.12568 | 0.51139 | 0.0012 | 0.00122 |
| 2.75 | 2.5375 | 0.11736 | 0.51352 | 0.0012 | 0.00121 |
| 3 | 2.2725 | 0.11736 | 0.50496 | 0.00108 | 0.00108 |
| 3.25 | 2.1267 | 0.10995 | 0.50823 | 0.0011 | 0.0011 |
| 3.5 | 1.98793 | 0.10603 | 0.5072 | 0.00107 | 0.00108 |
| 3.75 | 1.82149 | 0.10688 | 0.49672 | 9.4092E-4 | 9.48282E-4 |
| 4 | 1.69579 | 0.10764 | 0.48584 | 8.45091E-4 | 8.43842E-4 |
| 4.25 | 1.554 | 0.10848 | 0.4714 | 7.05143E-4 | 7.04082E-4 |
| 4.5 | 1.4587 | 0.10544 | 0.46254 | 6.06245E-4 | 6.09976E-4 |
| 4.75 | 1.37918 | 0.1003 | 0.45621 | 5.14112E-4 | 5.21393E-4 |
| 5 | 1.28935 | 0.09748 | 0.44545 | 4.16573E-4 | 4.18836E-4 |
| 5.25 | 1.23452 | 0.08705 | 0.44782 | 3.50832E-4 | 3.4353E-4 |
| 5.5 | 1.22646 | 0.06667 | 0.47214 | 3.15641E-4 | 2.90218E-4 |
| 5.75 | 1.09173 | 0.09457 | 0.41791 | 2.19884E-4 | 2.13824E-4 |
| 6 | 1.07894 | 0.07883 | 0.44054 | 1.8923E-4 | 1.81329E-4 |
| 6.25 | 1.0342 | 0.08087 | 0.44052 | 1.51884E-4 | 1.45139E-4 |
| 6.5 | 0.93537 | 0.1455 | 0.36024 | 1.21812E-4 | 1.22657E-4 |
| 6.75 | 0.87344 | 0.19946 | 0.28894 | 1.07052E-4 | 1.05249E-4 |
| 7 | 0.82622 | 0.23812 | 0.23295 | 9.25988E-5 | 9.14067E-5 |
| 7.25 | 0.79077 | 0.26323 | 0.19555 | 7.92166E-5 | 7.9873E-5 |
| 7.5 | 0.75304 | 0.29358 | 0.13718 | 6.82187E-5 | 7.01489E-5 |
| 7.75 | 0.72222 | 0.3169 | 0.09026 | 5.85398E-5 | 6.12053E-5 |
